# Case report: Peri-procedural hydroxyurea helps minimize bleeding in patients with Essential Thrombocythemia associated with acquired von Willebrand syndrome

**DOI:** 10.3389/fonc.2024.1326209

**Published:** 2024-02-01

**Authors:** Leah Kogan, Russell Price, Rouslan Kotchetkov

**Affiliations:** ^1^ Department of Medicine, University of Limerick, Limerick, Ireland; ^2^ Department of Pathology, Royal Victoria Regional Health Centre, Barrie, ON, Canada; ^3^ Simcoe Muskoka Regional Cancer Program, Royal Victoria Regional Health Centre, Barrie, ON, Canada

**Keywords:** Essential Thrombocythemia, von Willebrand syndrome, bleeding, hemorrhage, hydroxyurea

## Abstract

**Background:**

Essential Thrombocythemia is a chronic myeloproliferative neoplasm characterized by an isolated excessive production of platelets. Extreme thrombocytosis is defined by having a platelet count greater than or equal to 1,000 x 10^9^/L, which may lead to the development of acquired von Willebrand syndrome and complications of excessive hemorrhage.

**Case description:**

A 74-year-old female patient was brought in for a bone marrow examination regarding elevated platelet count. She had no history of excessive bleeding. The physical exam was unremarkable with no petechiae or hematomas. Complete blood count showed platelet count 1,491x10^9^/L. Bone marrow aspiration and biopsy were unremarkable, however, the patient developed bleeding from the biopsy site. Local pressure and an ice pack were ineffective, so she received 20 mcg of desmopressin subcutaneously, 1 unit of fresh frozen plasma and was started on tranexamic acid 1,000 mg orally every 8 hours. She was admitted for bleeding control and had another dose of desmopressin. Blood work showed elevated partial thromboplastin time and normal international normalized ratio. Acquired von Willebrand syndrome was suspected and a sample for von Willebrand disease was sent out. The next day her bleeding continued, and her Hb decreased from 145 to 89 g/L, she became symptomatic (tachycardic) and fatigued. The coagulation profile was consistent with acquired von Willebrand syndrome. Since she continued bleeding, she received 1 unit of packed red blood cells. A high dose of hydroxyurea (3g/day) was started urgently; within 24 hours platelet count was halved, and the bleeding resolved. Blood work was repeated 24 hours later and showed normalization of partial thromboplastin time and a normal Von Willebrand profile.

**Conclusion:**

Patients with extreme thrombocytosis are at high risk of bleeding due to acquired Von Willebrand Syndrome. Initiation of hydroxyurea at the time of bone marrow exam helps to control platelet count and minimizes the risk of peri-procedural hemorrhage in high-risk Essential Thrombocythemia patients with suspected acquired Von Willebrand Syndrome.

## 1 Introduction

Essential thrombocythemia (ET) is one of myeloproliferative neoplasms (MPN). It is a clonal hematopoietic stem cell disorder, characterized by an isolated overproduction of thrombocytes (1, 2). Disease severity and clinical features may depend on underlying molecular mutations. Mutations in Janus-activated kinase 2 (JAK2), myeloproliferative leukemia virus oncogene (MPL) and calreticulin (CALR) are the genes associated with ET. About 12% of patients are negative for all three mutations (triple negative), indicating the presence of additional not yet identified gene mutations (3). ET is associated with thrombotic complications and is a major cause of morbidity and mortality. Involvement of unusual venous sites (portal, hepatic, mesenteric veins) is often an initial presenting sign of ET. The goal of ET management is to reduce the risk of thrombotic complications. Treatment differs according to the risk stratification of ET, which is based on age, clinical factors history of thrombosis and JAK2 mutational status (4). Low-risk ET patients are usually on active surveillance or receive low-dose aspirin. High-risk patients also require cytoreductive therapy with either hydroxyurea (HU), anagrelide, and/or interferon-alpha (5). HU is effective in preventing thrombosis in high-risk ET patients (6).

Bleeding is less common in ET compared to thrombosis. It mainly affects mucosal sites, like nasal, or buccal mucosa. However, hemorrhage is also a well-known complication of ET, with median incidences of bleeding and major bleeding in 4.6 and 0.79% of patients per year. There is a wide range in bleeding risk reported across literature, ranging from 0.4-5.3% in patients with ET. Overall, compared to the general population the risk of bleeding is significantly elevated in patients with ET (7). Factors associated with a higher risk of bleeding include age >60, bleeding history, splenomegaly, myeloproliferative neoplasm subtype, and platelet count (8). Thus, clinical judgment is essential in estimating the risk of hemorrhage. Extreme thrombocytosis, defined as a platelet count greater than or equal to 1,000x10^9^/L, may lead to severe bleeding, due to acquired Von Willebrand syndrome (AVWS). Von Willebrand Factor (vWF) has an important role in protecting factor VIII from degradation and mediating platelet contact with injured vessel roles (9). Removal of the factor from plasma circulation, or its absorption into malignant cells leads to general low levels of vWF in AVWS, which in turn leads to variety in clinical presentation. In ET there is degradation of vWF via ADAMTS13, von Willebrand factor-cleaving protease, due to the high platelet count. Patients presenting with AVWS are at risk of experiencing heavy bleeds, in particular after invasive procedures (10).

## 2 Case presentation

### 2.1 Patient information

**Figure 1:**
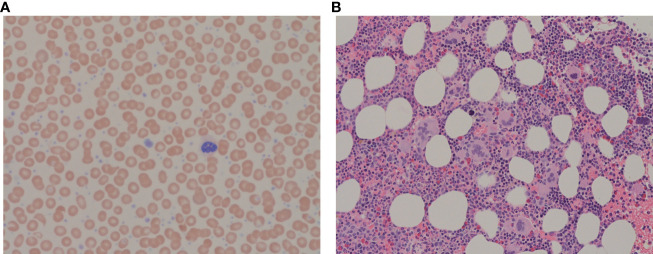
**(A)** Peripheral blood film showing increased platelets with clusters and giant platelets, morphologically normal white blood cells and erythrocytes. May-Giemsa, x63 objective. **(B)** Bone marrow trephine showing megakaryocytic clustering with atypical megakaryocytes. H&E, x20 objective.

A 74-year-old female patient was referred to our cancer center regarding elevated platelet count and suspected MPN. Her past medical history was significant for hypertension, and gastroesophageal reflux disease only. Home medications included perindopril 4 mg daily and, in the past, she was on omeprazole, stopped recently. She did not take anticoagulants or antiplatelet agents. There was no family history of excessive bleeding or coagulopathies. Her physical exam showed a body max index of 24.5, elevated systolic blood pressure (187/78 mm Hg), and no organomegaly, petechiae, or hematomas. Complete blood count showed normal hemoglobin (Hb) and white blood cell count, but platelet count was markedly elevated at 1,292x10^9^/L (**Figure 1A**). Partial Thromboplastin time (PTT) was 35 sec, at the upper limit of normal. International normalized ratio (INR) was 1.0 (normal). Biochemistry was unremarkable with normal lactic acid dehydrogenase and uric acid. Myeloproliferative neoplasm, in particular, Essential Thrombocythemia was suspected, and the patient was offered a bone marrow examination along with the molecular tests.

### 2.2 Diagnostic assessment/therapeutic intervention

**Figure 2:**
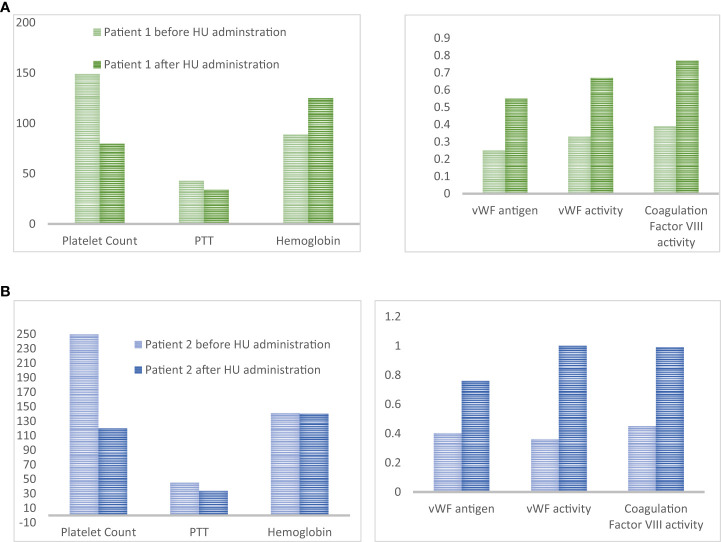
**(A)** Platelet count (x10^9^/L), PTT (seconds), Hemoglobin (g/L), vWF antigen (U/mL), vWF activity (U/mL) and coagulation factor VIII activity (U/mL) for patient #1 4 hours and 42 hours after bone marrow biopsy. Decrease platelet count after HU administration was accompanied by the normalization of coagulation parameters and von Willebrand panel. **(B)** Platelet count (x10^9^/L), PTT (seconds), Hemoglobin (g/L), vWF antigen (U/mL), vWF activity (U/mL) and coagulation factor VIII activity (U/mL). Decrease platelet count after HU administration was accompanied by the normalization of coagulation parameters and von Willebrand panel.

Due to personal reasons diagnostic bone marrow exam was delayed for two weeks. At that time her physical exam was unremarkable with no petechiae or hematomas. The procedure was technically uncomplicated, however, the patient developed bleeding from the biopsy site. Local pressure and an ice pack were ineffective, so 20 mcg of desmopressin subcutaneously, and one unit of fresh frozen plasma were given. Tranexamic acid 1,000 mg PO every 8 hours was also initiated. The patient was admitted for bleeding control and received another dose of desmopressin. As shown in **Figure 2** the patient had extreme thrombocytosis (platelet count rose to 1,491x10^9^/L), elevated PTT (43 sec), normal INR and Hb 145 g/L. A sample for testing for von Willebrand disease was sent out. The next day her bleeding continued and her Hb decreased to 89 g/L, she became symptomatic (tachycardia) and fatigued. The coagulation profile was consistent with AVWS type 2 subtype with a reduction in vWF: Ag: vWF Antigen 0.25 U/mL (normal 0.56-1.62), vWF Activity was 0.33 U/mL (normal 0.50-1.87), Coagulation Factor VIII Activity was 0.39 U/mL (normal 0.60-1.71. The patient’s blood group was O-negative with a negative antibody screen.

She received one unit of packed red blood cells and a high dose of HU (3g/day) was started urgently; within 24h platelet count dropped to 798x10^9^/L and the bleeding resolved. Repeated blood work showed normalization of PTT (34 sec) and vWF profile (**Figure 2**). The dose of HU was decreased to 500 mg PO every 12 hours. She was seen in follow-up in 2 weeks. At that time Hb was 125 g/L, reticulocyte count was appropriately elevated (128), and she developed iron deficiency. The platelet count was down to 450x10^9^/L. She started on oral iron supplements and the HU dose was further decreased to 500 mg daily. She started aspirin and had no further bleeding. Diagnosis of ET was confirmed on a bone marrow biopsy. It showed hypercellular bone marrow with clusters of abnormal megakaryocytes (**Figure 1B**). The molecular test was positive for JAK2 V617F mutation in both alleles. She remained on this dose of HU for several years.

## 3 Discussion

### 3.1 Awareness about AVWS

AVWS, defined as an acquired deficiency or dysfunction of vWF, is much less common, compared to inherited von Willebrand disease. It is not uncommon that the diagnosis of AVWS is overlooked in a bleeding patient. This is in part due to asymptomatic presentation, and manifestations of symptoms in conjunction with another disorder. AVWS is most typically caused by a combination of several mechanisms. Bleeding symptoms in an individual with any of the diseases summarized in **Table 1** should raise suspicion and prompt an evaluation for AVWS. The etiology of AVWS is an important factor when considering therapy, as it allows for the design of a targeted treatment plan (15).

**Table 1:** Summary of underlying disease contributing to AVWS and mechanism of pathology.

Mechanism	Associations	Resemblance to congenital vWF	Reference
Formation of antibodies targeting vWF in an autoimmune reaction either directly or through competitive inhibition.	Multiple myeloma, erythematosus lupus, autoimmune disorders, endocrine	N/A	(11–13)
Proteolysis of vWF in response to shear forces	Ventricular septal defects, aortic stenosis, primary pulmonary hypertension	Type 2a	(12)
Increased vWF adhesion to other cell surfaces preventing functionality.	Myeloproliferative disorders	N/A	(12, 13)
Decreased vWF synthesis	Hypothyroidism	Type 1	(13, 14)

vWF, Von Willebrand Factor; N/A, Not applicable.

AVWS has been seen in all of the MPNs but is most commonly found in association with ET. A retrospective analysis of 170 patients with ET who had laboratory testing, found concomitant AVWS in 34 patients (20%). AVWS was diagnosed on the basis of reduced levels of vWF and abnormal results of other routine tests (16). Higher platelet count was associated with AVWS, though only three (1.8%) had platelet counts >1,000x10^9^/L. The mechanism of AVWS in ET patients is not fully understood, and as such patients with AVWS may present with either high or non-elevated platelet counts. Such patients are often missing higher molecular weight multimers of vWF, suggesting increased proteolysis by platelet proteases. Another proposed mechanism includes the dysfunction of platelets which could explain AVWS in patients with low platelet counts (7). Markedly increased numbers of platelets alone may bind a higher quantity of vWF, allowing vWF to unfold and become susceptible to ADAMTS13, the primary enzyme that cleaves vWF in the circulation (17). In addition, increased vWF adhesion to other cell surfaces prevents platelet functionality (12, 13). The complex pathophysiology of AVWS combined with the predominant asymptomatic presentation makes it difficult to diagnose AVWS, and thus be aware of possible complications. AVWS can lead to both thrombotic and hemorrhagic complications, and extreme thrombocytosis is not always present (7, 16).

The first step in the management of ET with underlying AVWS is risk stratification and screening for AVWS in high-risk patients. Screening of patients with ET and other MPNs should be a first-line step (14). Patients presenting with younger age, JAK2 mutation, high Hb, high hematocrit, high platelet count, and mucocutaneous bleeding tend to have an increased chance of having AVWS (7, 14). In our case extreme thrombocytosis was present, however, platelets greater than 1,000x10^9^/L may not be necessary to develop AVWS. In patients with ET, a high hematocrit is indicative of high blood viscosity and can lead to increased sheer stress and further to increased destruction of high molecular weight multimers and an increased risk of AVWS development (18). Thus ET patients with platelet counts of greater than 1,000x10^9^/L with high hematocrit should be assessed for AVWS. The complex pathophysiology, and asymptomatic presentation of AVWS means that one should not discount patients with comorbidities of ET and AVWS presenting with platelet counts less than 1,000x10^9^/L.

Management of AVWS is complicated when associated with ET, where the risk of bleeding and management of it, is hindered by the present risk of a thrombotic event (10). The typical treatment of ET being an antiplatelet therapy poses an additional risk for patients with ET. When preparing a patient for an invasive procedure, with a risk of bleeding, screening for AVWS can reduce the risk of complication, and allow the clinician to anticipate the potential for bleeding. Patients with a JAK2 mutation are overall at higher risk of developing AVWS as well as subsequent complications. When compared to CALR-mutated patients JAK2 patients have more activated adenosine diphosphate (ADP), an indication of higher platelet activity. Overall CALR-mutated patient with ET has a better prognosis when compared to JAK2 (19).

### 3.2 Risk assessment for venous thromboembolism and hemorrhage prior to bone marrow biopsy

The balance of thrombotic versus hemorrhagic complications heavily depends on the underlying AVWS cause. There is not a significant correlation between a JAK2 mutation and increased incidence of hemorrhagic complications, however major and minor bleeding incidence did occur in 8.7 to 11.6 percent of patients respectively (18)

With an MPN as an underlying cause, there is a three-fold risk of a bleeding complication when compared to a lymphoproliferative disease (10). As the management of high-risk ET includes low-dose aspirin, there is a high correlation between aspirin use and bleeding incidence (10, 20). As such, it is important to ensure that ET patients with high risk for bleeding are not on any anti-coagulants, or aspirin prior to undergoing a procedure such as bone marrow biopsy.

Since the primary objective of ET management is to prevent thromboembolic complications, the risk of venous thromboembolism must be assessed in each ET patient. The International prognostic score of thrombosis in Essential Thrombocythemia (IPSET-Thrombosis) model provides objective estimates of the probability of thrombotic events in patients with newly diagnosed ET (21). Scores of ≥3 points put patients into the high-risk category with an annual thrombosis risk of 3.56% (4). The revised IPSET-thrombosis identified four risk categories based on three adverse variables (thrombosis history, age >60 years and JAK2V617F): very low (no adverse features), low (presence of JAK2V617F), intermediate (age >60 years) and high (presence of thrombosis history or presence of both advanced age and JAK2V617F) (22). Recently, it was shown that the IPSET model can also be used to predict hemorrhagic complications (23).

As previously recommended, patients with extreme thrombocytosis over 1,000x10^9^/L should be tested for AVWS. Aspirin should be held if vWF: RCoA<30% or there is the absence of high molecular weight multimers (10).

### 3.4 Peri-procedural management of ET patients with a high risk of bleeding

When AVWS presents in combination with ET the treatment goal is to reduce platelet count and normalize their function. Approximately 10% of patients with ET undergoing surgical interventions can experience bleeding, and about 7% of cases are deemed to be a major bleeding event (7).

There is no complete standardization for AVWS treatment, as such standard management strategies for AVWS are reactive to bleeding or thrombotic events. The first step is to apply a local pressure pad or tourniquet and ice. If the bleeding continues, the next step is the administration of desmopressin, also known as 1-desamino-8-D-arginine vasopressin. It is an analogue of vasopressin, which stimulates vWF release through V2 receptor activation. In congenital vWF disease, desmopressin is most effective in types 1 and type 2 (A, N, M), while having no therapeutic benefit in type 3. This occurs due to the total absence of vWF, thus stimulation of endothelial cells to release vWF is ineffective (24). AVWS treatment depends on the severity of symptoms but can also be initiated prophylactically prior to a procedure. Unfortunately, desmopressin is less effective in AVWS severe forms of both type 1 and 2 (A, N, M) and completely contraindicated in type 2B vWF disease (12, 24, 25). Treatment of AVWS with desmopressin provides only short-term relief but often has re-occurrence of hemorrhagic events. Desmopressin is most effective in treating thrombotic complications of AVWS, whereas with hemorrhagic complications it shows poor efficacy (26) In fact, desmopressin showed only a 30% success rate, with even lower rates when the underlying disorder was cardiovascular or MPN in nature (14). Antifibrinolytic agents (tranexamic acid) can be used as an adjunct to other therapies. Other therapy options include vWF/FVIII infusions, IVIG infusion, and anti-fibrinolytics. The variety of underlying diseases makes standard treatment difficult for AVWS patients (14).

**Figure 3:**
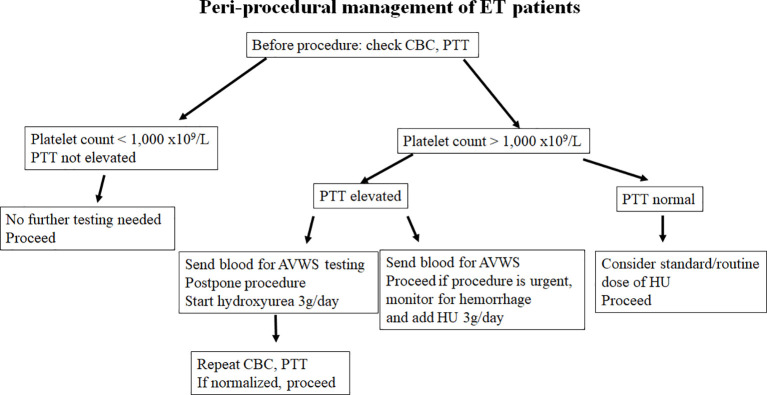
Flow chart showing our suggested risk stratification and management of patients presenting with extreme thrombocytosis, concurrent with ET and AVWS.

Our case shows that the addition of HU can act as a fast, and effective cytoreductive method in reducing bleeding risk in ET patients with extreme thrombocytosis and suspected AVWS. HU induces replication stress which arrests megakaryocytes in the S-phase of the cell cycle. It does so by inhibiting the activity of ribonucleotide reductase which prevents the synthesis of new DNA. Dysfunction in the S phase of the cell cycle activates a checkpoint which arrests the cell in this cycle until the DNA damage can be fixed, if the DNA damage cannot be fixed the cell undergoes apoptosis. In this way the activation of the S-phase checkpoint leads to decreased number of platelet precursors, thus reducing thrombocytosis and restoring platelet functionality (27). Recently we had another similar case of patients with suspected, but not yet confirmed ET (platelet count > 2,500x10^9^/L) and possible AVWS (PTT was 45 sec with upper limit of normal being 35, work up for vWD was underway). We postponed diagnostic bone marrow aspiration and biopsy and preventively added a high dose of HU (3g daily) x3 days. On the day of the bone marrow biopsy, the platelet count was down to 1,200 and PTT returned to normal (34 sec). Prophylactic use of HU allowed for the go-ahead with the bone marrow examination, which was completed without the occurrence of a hemorrhagic event. Retrospectively, the patient was found to have AWVS before (vWF Antigen 0.4 U/mL (normal 0.56-1.62), vWF activity was 0.36 U/mL (normal 0.50-1.87), coagulation Factor VIII Activity was 0.45 U/mL (normal 0.60-1.71) (**Figure 2B**). All parameters normalized by the time of bone marrow examination. Based on our experience, the significant cytoreductive effect induced by urgent administration of high doses of HU was able to provide adequate hemorrhage control. We suggest that HU may be an underused tool to control platelet count in patients who developed post-procedure bleeding. Moreover, the same strategy could also be adopted in patients with platelet counts within 1,000 x10^9^/L, but in the presence of other risk factors for AVWS. Alternatively, high-dose HU can be started prophylactically, two to four days prior to the planned procedure to decrease the likelihood of post-procedure bleeding, as proposed in **Figure 3**. A short course of high-dose HU prior to bone marrow biopsy will be unlikely to change the bone marrow morphology but will drastically decrease the patient’s risk of bleeding.

## 4 Conclusion

Extreme thrombocytosis is a risk factor for bleeding. Identification of risk factors for both thrombosis and bleeding are important. Patients with extreme thrombocytosis should be screened for AVWS. However, presence of other risk factors such as JAK2 mutation, high Hb, high hematocrit, platelet count, and mucocutaneous bleeding who tend to have an increased chance of having AVWS, should also be monitored for possible development of complications. They require additional monitoring and should have a low threshold to intervene to control bleeding. In addition to the standard treatments for hemorrhagic complications, we recommend consideration of a short course of high-dose HU initiation as a fast, and effective method of achieving control of acute bleeding prior to invasive procedures, including bone marrow aspirations and biopsy.

## Data availability statement

The original contributions presented in the study are included in the article/supplementary material. Further inquiries can be directed to the corresponding author.

## Ethics statement

Ethical approval is not required for this study in accordance with our local guidelines. The research was conducted ethically in accordance with the World Medical Association Declaration of Helsinki. Written informed Consent for Publication was obtained from the patient for publication of the details of their medical case and any accompanying images.

## Author contributions

LK: Conceptualization, Formal analysis, Investigation, Methodology, Writing – original draft, Writing – review & editing. RP: Methodology, Visualization, Writing – original draft, Writing – review & editing. RK: Conceptualization, Methodology, Resources, Supervision, Writing – original draft, Writing – review & editing.
